# Leaf Gas Exchange Performance of Ten Quinoa Genotypes under a Simulated Heat Wave

**DOI:** 10.3390/plants9010081

**Published:** 2020-01-09

**Authors:** Ashley Eustis, Kevin M. Murphy, Felipe H. Barrios-Masias

**Affiliations:** 1 Department of Agriculture, Veterinary and Rangeland Sciences, College of Agriculture, Biotechnology and Natural Resources, University of Nevada, Reno, NV 89557, USA; ajeustis@gmail.com; 2 Department of Crop and Soil Sciences, College of Agricultural, Human, and Natural Resource Sciences, Washington State University, Pullman, WA 99164-6420, USA; kmurphy2@wsu.edu

**Keywords:** *Chenopodium quinoa*, heat stress, chlorophyll fluorescence, photosynthesis, dark respiration

## Abstract

Quinoa (*Chenopodium quinoa* Willd.) is a highly nutritious crop that is resilient to a wide range of abiotic stresses; however, sensitivity to high temperatures is regarded as an impediment to adoption in regions prone to heat waves. Heat stress is usually associated with a decrease in crop reproductive capacity (e.g., pollen viability), yet little is known about how leaf physiological performance of quinoa is affected by high temperatures. Several trials were conducted to understand the effect of high temperatures, without confounding stressors such as drought, on ten selected quinoa genotypes considered to encompass heat sensitive and heat tolerant plant material. Plants were grown under favorable temperatures and exposed to two temperature treatments over four consecutive days. The heat treatment simulated heat waves with maximum and minimum temperatures higher during the day and night, while the control treatment was maintained under favorable temperatures (maximum and minimum temperatures for ‘Heat’: 45/30 °C and ‘Control’: 20/14 °C). Leaf gas exchange (day), chlorophyll fluorescence (predawn and day) and dark respiration (night) were measured. Results show that most quinoa genotypes under the heat treatment increased their photosynthetic rates and stomatal conductance, resulting in a lower intrinsic water use efficiency. This was partly corroborated by an increase in the maximum quantum yield of photosystem II (F_v_/F_m_). Dark respiration decreased under the heat treatment in most genotypes, and temperature treatment did not affect aboveground biomass by harvest (shoot and seeds). These results suggest that heat stress alone favors increases in leaf carbon assimilation capacity although the tradeoff is higher plant water demand, which may lead to plant water stress and lower yields under non-irrigated field conditions.

## 1. Introduction

Quinoa (*Chenopodium quinoa* Willd.) is a highly nutritious and stress tolerant crop that has gained attention in the efforts to address food security under the effects of global warming and projected population growth [1,2]. Native to the Andean region of South America, quinoa is a diverse crop species divided into five globally recognized ecotypes, each adapted to unique conditions resulting in tolerance to many abiotic stressors [2,3]. Quinoa grows from sea level to >4500 meters above sea level in environments with high irradiance, freezing temperatures, and water deficit stress, suggesting excellent potential as an alternative crop for growers adapting to climate change [4,5]. The projected increase in global temperatures, approximately 1 °C and 3 °C above the present value by 2025 and 2100, respectively, have increased the pressure to gain a better understanding of plant responses to heat stress [6,7,8]. Quinoa sensitivity to high temperatures is regarded as an impediment to adoption in regions prone to heat waves [9], and a better understanding of leaf physiological responses to temperature can help breeding efforts for new cultivars with better performance under heat stress.

High temperature stress in plants has a negative impact on physiological and biochemical processes, depending on the length of exposure to extreme temperatures [10]. In general, a temporary (hours to days) rise in temperature of 10–15 °C above ambient is considered heat stress, but even plants exposed to temperatures 5 °C higher than normal can experience stress and reduced growth [6,11]. Heat stress has been reported as one of the most important causes of reductions in biomass and yield in many crops, including maize (*Zea mays*), common bean (*Phaseolus vulgaris*), wheat (*Triticum aestivum*), and quinoa [9,10,11]. Plant species and even genotypes respond differently to heat stress, and their performance under extreme temperatures partly depends on the capacity of the leaf to maintain carbon (C) assimilation.

Plant susceptibility to high temperatures varies with developmental stage, which will dictate the degree of possible damage incurred at vegetative and reproductive stages [6,11]. High temperatures affect enzymatic reactions (i.e., Rubisco kinetics), and the capacity of a leaf to acclimate to heat can depend on a lower activation state of Rubisco at higher temperatures, which will affect its photosynthetic capacity [12]. In general, heat stress induced responses in plants include modifications to the photosynthetic machinery, organizational changes in cellular structures to maintain membrane functions, and stomatal closure to reduce transpirational water loss [6,13,14]. Structural changes due to heat stress occur in chloroplast–protein complexes, with the chloroplast stroma and thylakoid membranes considered the primary sites of heat-induced damage. These changes are accompanied with a loss of grana stacking, reduced enzymatic activity and ion leakage due to membrane damage [15,16,17,18], resulting in damage to photosystem-II (PSII) and a reduction in the leaf photosynthetic capacity. This damage can be quantified by assessing gas exchange and chlorophyll fluorescence parameters, in particular the maximum quantum yield of PSII (F_v_/F_m_), which has optimal values around 0.83 for most plant species [19,20]. Additional decreases in photosynthetic efficiency can be due to photoinhibition or increased photorespiration as Rubisco affinity for O_2_ increases and solubility and diffusion of CO_2_ declines [12]. In addition, leaf respiration is also a sensitive process to high temperatures, and generally, more photosynthates are used for general maintenance under stress. A reduction in photosynthesis and net carbon assimilation will eventually result in limited resource availability for reproduction and an overall decrease in plant biomass [6,10,21,22,23,24,25].

While there is a large body of research on crop responses to heat stress (e.g., focused on pollen viability or confounded with other stressors), there are limited studies available on quinoa leaf responses to solely extreme high temperatures. The present study aimed to understand the effects of high temperature on leaf gas exchange when quinoa plants were exposed to a four-day heat wave without drought as a confounding effect. We measured 10 quinoa genotypes, selected from a previous screening of 112 lines evaluated under high temperature conditions [26] for several parameters, including photosynthetic rate, stomatal conductance, intrinsic water use efficiency (WUE_i_), chlorophyll fluorescence, membrane stability, aboveground biomass (shoots and seeds), and seed biomass.

## 2. Results

### 2.1. Photosynthetic Rate 

**Figure 1:**
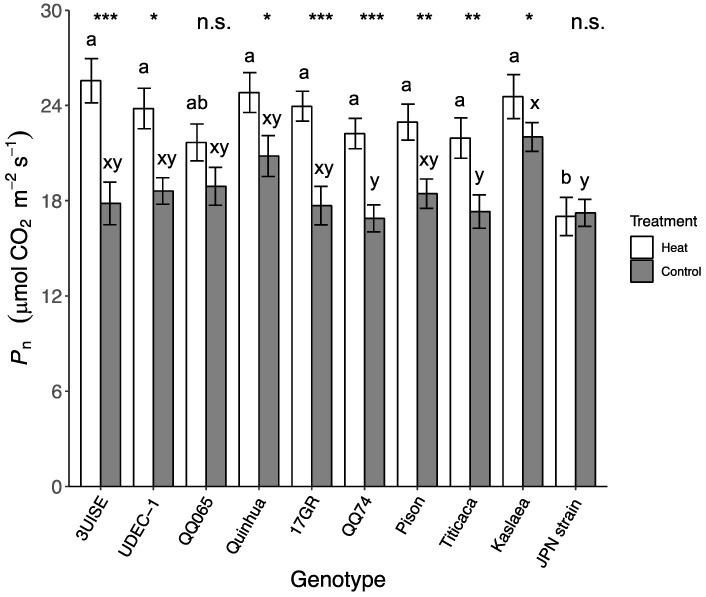
Photosynthetic rate (P_n_; μmol CO_2_ m^−2^ s^−1^) of 10 quinoa genotypes exposed to a four-day heat treatment (45 °C/30 °C) and control treatment (20 °C/14 °C); day and night, respectively. Values are means ± standard error (SE) (*n* = 32). Mean comparisons are shown for within genotype (asterisks and n.s.) and within temperature treatment (Heat: a to b; Control: x to y); different letters are significantly different at *p* < 0.05; *, *p* < 0.05; **, *p* < 0.01; ***, *p* < 0.001; n.s., not significantly different.

The photosynthetic rate (P_n_) in quinoa showed an interaction between the temperature treatments and genotypes. Overall, the P_n_ was higher in the heat treatment compared to the control for all genotypes except for QQ065 and Japanese Strain (Figure 1). In the heat treatment, Japanese Strain had at least a 22% lower P_n_ than the other genotypes except for QQ065. The P_n_ of all other genotypes were similar and ranged between 22.0 and 25.6 μmol CO_2_ m^−2^ s^−1^. Within genotype, 3UISE had the highest increase in P_n_ (44%) under the heat treatment compared to the control. Kaslaea showed only a 12% increase in P_n_, but within the control treatment, this genotype had one of the highest P_n_ rates. In the control treatment, the P_n_ of Kaslaea was 30%, 28%, and 27% higher than QQ74, Titicaca and Japanese Strain, respectively. No other differences in P_n_ were observed.

### 2.2. Stomatal Conductance

**Figure 2:**
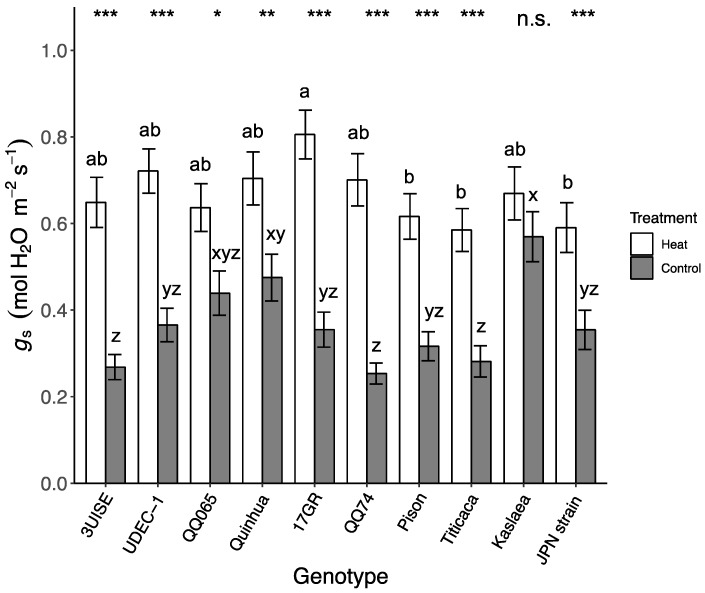
Stomatal conductance (g_s_; mol H_2_O m^−2^ s^−1^) of 10 quinoa genotypes exposed to a four-day heat treatment (45 °C/30 °C) and control treatment (20 °C/14 °C); day and night, respectively. Values are means ± SE (*n* = 32). Mean comparisons are shown for within genotype (asterisks and n.s.) and within temperature treatment (Heat: a to b; Control: x to z); different letters are significantly different at *p* < 0.05; *, *p* < 0.05; **, *p* < 0.01; ***, *p* < 0.001; n.s., not significantly different.

Stomatal conductance (g_s_) showed an interaction between genotype and temperature similar to observations for P_n_. Overall, g_s_ was higher in the heat treatment group compared to the control for all genotypes except Kaslaea, which had one of the highest g_s_ values under the control treatment (Figure 2). Under the heat treatment, 3UISE, 17GR, QQ74 and Titicaca had over a 100% increase in g_s_ compared to the control treatment, while UDEC-1, QQ065, Quinhua, Pison and Japanese Strain showed increased g_s_ values ranging from 45% to 95% relative to the control. Within the heat treatment group, 17GR had one of the highest g_s_ values, which was 40%, 37% and 31% higher than Titicaca, Japanese Strain and Pison, respectively, which had the lowest g_s_ values. Within the control treatment group, Kaslaea and Quinhua had among the highest g_s_ values, which were 104% and 64% higher than QQ74, 3UISE and Titicaca, which had the lowest g_s_ values.

### 2.3. Intrinsic Water Use Efficiency

**Figure 3:**
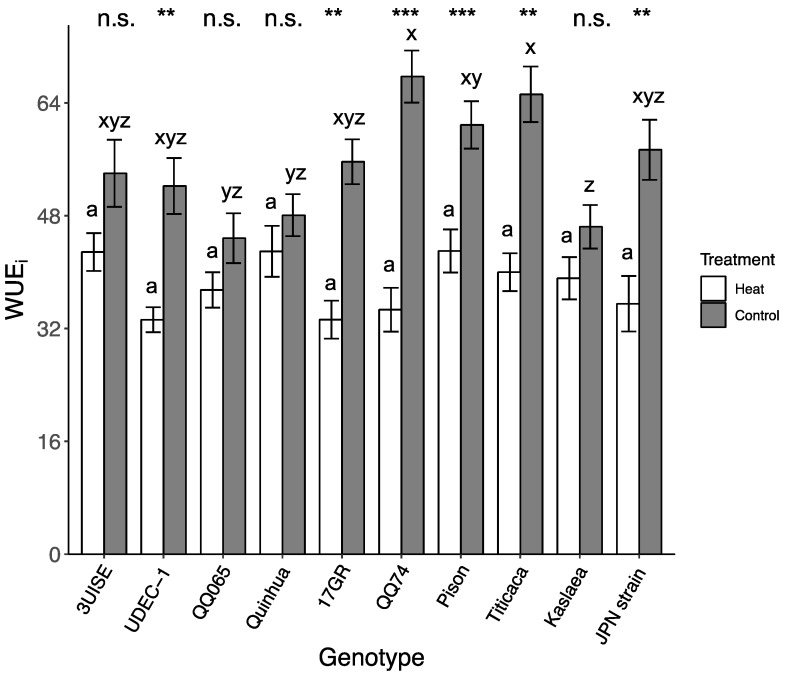
Intrinsic water use efficiency (WUE_i_; calculated as P_n_/g_s_) of 10 quinoa genotypes exposed to a four-day heat treatment (45 °C/30 °C) and control treatment (20 °C/14 °C); day and night, respectively. Values are means ± SE (*n* = 23–32). Mean comparisons are shown for within genotype (asterisks and n.s.) and within temperature treatment (Heat: a; Control: x to z); different letters are significantly different at *p* < 0.05; *, *p* < 0.05; **, *p* < 0.01; ***, *p* < 0.001; n.s., not significantly different.

Intrinsic water use efficiency (WUE_i_) also reflected an interaction between genotype and temperature. WUE_i_ decreased in the heat treatment group for the majority of the genotypes except for 3UISE, QQ065, Quinhua and Kaslaea, which showed no change compared to the control treatment (Figure 3). In the heat treatment group, WUE_i_ was similar among genotypes, and it ranged from 33.2 ± 1.78 (UDEC-1) to 43.0 ± 3.06 (Pison). In the control group, WUE_i_ rates ranged between 46.4 ± 3.08 (Kaslaea) and 68.1 ± 3.78 (QQ74). In the control treatment group, Kaslaea had one of the lowest WUE_i_ values and was 31%, 40% and 46% lower than Pison, Titicaca and QQ74. Within genotypes, the WUE_i_ values of QQ74, UDEC-1, 17GR, Pison, Titicaca and Japanese Strain in the heat treatment group decreased between 29% and 49% compared to the control treatment.

### 2.4. Dark Respiration

**Figure 4:**
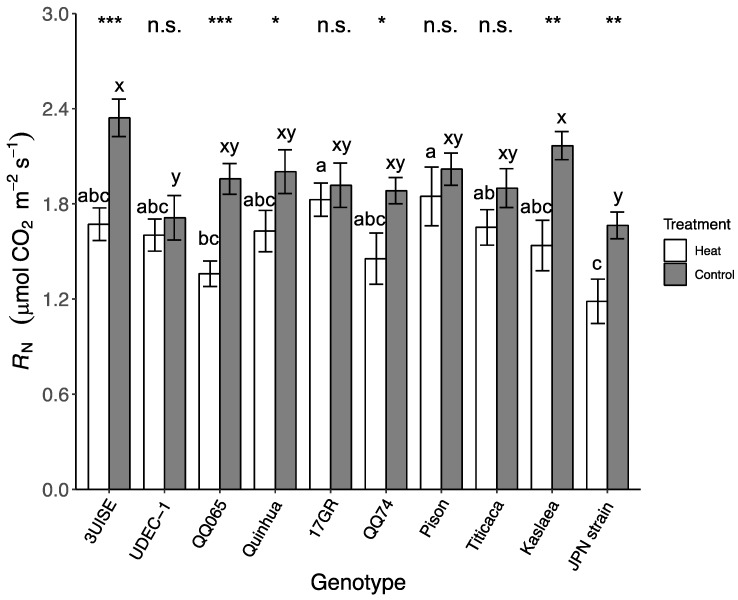
Dark respiration (R_N_; μmol CO_2_ m^−2^ s^−1^) of 10 quinoa genotypes exposed to a four-day heat treatment (45 °C/30 °C) and a control treatment (20 °C/14 °C); day and night, respectively. Measurements were taken in full darkness between 21:30 and 23:00. Values are means ± SE (*n* = 18–28). Mean comparisons are shown for within genotype (asterisks and n.s.) and within temperature treatment (Heat: a to c; Control: x to y); different letters are significantly different at *p* < 0.05; *, *p* < 0.05; **, *p* < 0.01; ***, *p* < 0.001; n.s., not significantly different.

Dark respiration (R_N_) showed a significant interaction between treatment and genotype, as with the other leaf gas exchange parameters. For most genotypes, R_N_ decreased under heat treatment compared to the control, except for UDEC-1, 17GR, Pison and Titicaca (Figure 4). The decrease in R_N_ for the heat treatment compared to the control ranged between 19% and 31%. R_N_ in the heat treatment was between 1.19 ± 0.14 μmol CO_2_ m^−2^ s^−1^ (Japanese Strain) and 1.85 ± 0.18 μmol CO_2_ m^−2^ s^−1^ (Pison). Within the heat treatment, Pison and 17GR had among the highest R_N_, which were approximately 35% higher than QQ065 and approximately 55% higher than Japanese Strain, which had among the lowest R_N_. Within the control treatment, 3UISE and Kaslaea had some of the highest R_N_, and were between 27% and 41% higher than UDEC-1 and Japanese Strain, respectively, which were possibly some of the lowest.

### 2.5. Maximum Quantum Yield of Photosystem-II

**Figure 5:**
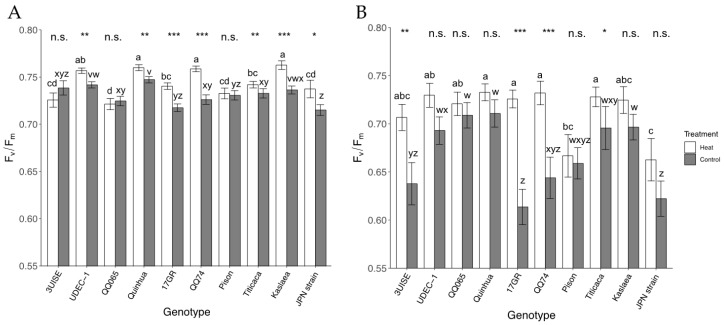
Maximum quantum yield of photosystem II (F_v_/F_m_) measured at pre-dawn (**A**) and afternoon (**B**) of 10 quinoa genotypes exposed to a four-day heat treatment (45 °C/30 °C) and control treatment (20 °C/14 °C); day and night, respectively. Measurements were taken between 05:00 and 06:30 (pre-dawn) and between 14:00 and 15:30 (afternoon). Values are means ± SE (*n* = 15–32). Mean comparisons are shown for within genotype (asterisks and n.s.) and within temperature treatment (Heat: a to d; Control: w to z); different letters are significantly different at *p* < 0.05; *, *p* < 0.05; **, *p* < 0.01; ***, *p* < 0.001; n.s., not significantly different.

Pre-dawn F_v_/F_m_ also showed an interaction between treatment and genotype. Although small changes in pre-dawn F_v_/F_m_ were observed, most genotypes in the heat treatment group had higher pre-dawn F_v_/F_m_ than the controls, except 3UISE, QQ065 and Pison (Figure 5A). The heat treatment F_v_/F_m_ ranged between 0.721 ± 0.006 and 0.763 ± 0.005 across all genotypes. The highest F_v_/F_m_ were observed in UDEC-1, QQ74, Quinhua and Kaslaea, while the lowest value was observed in QQ065. Within the control treatment, F_v_/F_m_ ranged between 0.7215 ± 0.006 and 0.747 ± 0.003 across genotypes. Similar to the heat treatment, Quinhua, UDEC-1, and Kaslaea had the highest F_v_/F_m_. The Japanese Strain was lower than all genotypes except 17GR and Pison in the control treatment.

Afternoon F_v_/F_m_ also showed an interaction effect between genotype and treatment (Figure 5B). In the heat treatment, the afternoon F_v_/F_m_ were higher in 3UISE, 17GR, QQ74 and Titicaca than their respective controls; a 20%, 14%, 11% and 4.3% increase, respectively, in the heat treatment group compared to the controls. Within the heat treatment group, Japanese Strain was between 8.3% and 10.6% lower than most genotypes except Pison, 3UISE, and Kaslaea. Within the control, 17GR and Japanese Strain had among the lowest F_v_/F_m_ and were at least 11.6% and 10.0% lower than UDEC-1, QQ065, Quinhua, Titicaca and Kaslaea.

### 2.6. Relative Chlorophyll Content

Overall, the relative chlorophyll content was affected by temperature through the course of the four-day treatment (*p* < 0.01; Figure S1). The genotypes 17GR, Pison and Titicaca tended to increase their chlorophyll content during the heat treatment (i.e., positive slope) compared to the control, where chlorophyll tended to decrease during the four-day treatment (i.e., negative slope) (*p* < 0.02). The rest of the genotypes did not show a significant change in chlorophyll content between the heat and the control treatments. In the heat treatment, all genotypes showed a positive slope except for Japanese Strain, which showed a 9.8% decrease between Day 1 (35.18 ± 5.21) and Day 4 (31.71 ± 5.61), and in the control treatment, all genotypes showed a negative slope except for Quinhua and QQ74.

**Figure 6:**
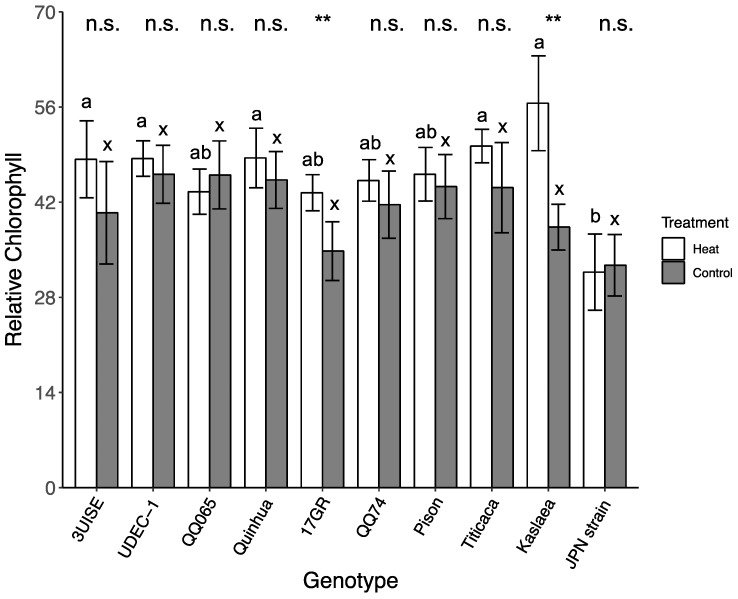
Relative chlorophyll for Day 4 in 10 quinoa genotypes exposed to a four-day heat treatment (45 °C/30 °C) and control treatment (20 °C/14 °C); day and night, respectively. Measurements were taken midafternoon between 14:00 and 15:30. Values are means ± SE (*n* = 5–9). Mean comparisons are shown for within genotype (asterisks and n.s.) and within temperature treatment (Heat: a to b; Control: x); different letters are significantly different at *p* < 0.05; *, *p* < 0.05; **, *p* < 0.01; ***, *p* < 0.001; n.s., not significantly different.

By the end of the temperature treatment (Day 4), 17GR and Kaslaea had a higher relative chlorophyll content in the heat treatment group compared to the control (24.7% and 47.5% increase, respectively) (Figure 6). Within the heat treatment group, Japanese Strain had the lowest relative chlorophyll content, and was at least 52% lower than 3UISE, Quinhua, Titicaca and Kaslaea, which had the highest values. Within the control treatment, no significant differences were found among genotypes.

### 2.7. Seed and Shoot Biomass

**Figure 7:**
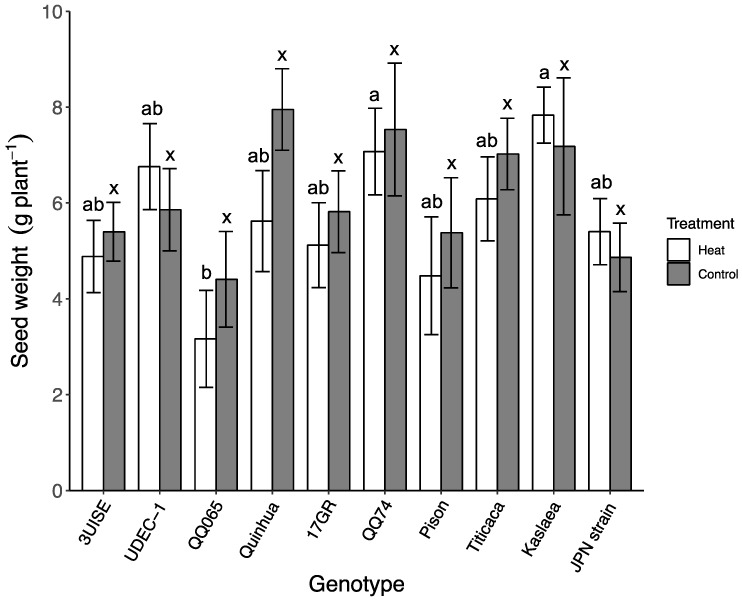
Seed weight (g plant^−1^) in 10 quinoa genotypes exposed to a four-day heat treatment (45 °C/30 °C) and control treatment (20 °C/14 °C); day and night, respectively. Values are means ± SE (*n* = 10–12). Mean comparisons are shown for within temperature treatment (Heat: a to b; Control: x); different letters are significantly different at *p* < 0.05. No differences were found for temperature treatment within genotype.

Seed biomass (as measured by seed weight grams per plant) was not affected by the temperature treatment, and differences were only observed among genotypes within the heat treatment (Figure 7). There was no interaction between genotype and treatment. Seed biomass ranged between 4.40 to 7.90 g plant^−1^ for the control treatment, and 3.20 to 7.80 g plant^−1^ for the heat treatment. Quinhua was the only genotype to show a trend towards lower seed biomass in the heat treatment group compared to the control (*p* = 0.06). Within the heat treatment, Kaslaea and QQ74 had the highest seed biomass, which was 148% and 124% higher than QQ065, which was one of the lowest. UDEC-1 tended to have a higher seed biomass than QQ065 in the heat treatment group (*p* = 0.07). Aboveground biomass (i.e., shoots and seeds) was not affected by genotype or temperature treatment, and biomass was on average 14.4 ± 0.5 g plant^−1^ for the control and 14.9 ± 0.5 g plant^−1^ for the heat treatment (data not shown).

## 3. Discussion

This study shows that quinoa can withstand exposures to a simulated heat wave with temperatures as high as 45 °C for a period of four days when no other confounding stressors such as drought are present. Under well-watered conditions, leaf gas exchange in quinoa was not sensitive to high temperatures, and in most genotypes, our data showed an increase in P_n_ and g_s_. The observed enhanced performance in C assimilation capacity was supported by an increased efficiency in the maximum quantum yield of PSII (i.e., F_v_/F_m_) under the heat treatment. The latter may be associated with the observed increase in relative chlorophyll content during the period that plants were exposed to elevated temperatures. Interestingly, R_N_ decreased under heat treatment conditions, which did not correlate with the increase in C assimilation capacity. In addition, aboveground biomass (shoots and seeds) and seed biomass were unaffected by the temperature treatments. It has been assumed that the cultivation of quinoa in the northern hemisphere has been constrained by its sensitivity to heat stress [9], similar to the impact of temperature on other seed producing crops (e.g., sorghum) [27,28]. Yet, our data shows that for most of the 10 evaluated genotypes, high temperature improved quinoa’s capacity for carbon assimilation and had no negative effect on seed production when soil moisture was not limiting.

Leaf gas exchange responses of quinoa to elevated temperatures (40 °C) have been shown to be similar to responses in control temperatures [29] and lowered when exposed to confounding stressors such as drought [30] and salinity [31]. Many other crops such as soybean (*Glycine max*), wheat, sorghum (*Sorghum bicolor*), rice (*Oryza sativa*), tobacco (*Nicotiana tabacum*) and grapevine (*Vitis vinifera*) show reductions in photosynthetic parameters when exposed to elevated temperatures (at least 5 °C above optimum) [6,10,23,32]. These reductions in C assimilation capacity may result from damage to the photosynthetic apparatus, particularly the thylakoid membranes, where PSII is located [10]. Additionally, P_n_ and g_s_ can be inhibited due to decreases in the activation state of Rubisco as a result of heat stress [6,10,23]. Our data suggests that some quinoa genotypes can even improve their C assimilation capacity as observed from increases in F_v_/F_m_, which implies no damage to PSII even at temperatures of 45 °C.

Under high temperatures, soil water availability and increased g_s_ can maintain evaporative cooling [23], but this may result in lower WUE_i_ when changes in P_n_ are proportionally smaller than fluctuations in g_s_. For instance, g_s_ in cotton increased under elevated temperatures, resulting in lower leaf temperatures. Typically, these heat avoidance mechanisms require an ample water supply, which results in a reduction in WUE_i_ [23,33,34]. In this study, the heat treatment generally resulted in lower WUE_i_ than the control with the exception of two genotypes. For instance, Quinhua increased both P_n_ and g_s_, and Kaslaea already had a high g_s_ under the control treatment, resulting in a low WUE_i_ under both temperature treatments.

The observed increases in leaf gas exchange were accompanied by a higher F_v_/F_m_ under high temperatures. Decreases in F_v_/F_m_ compared to non-stressed conditions usually indicate an impaired capacity for electron transport in the photosynthetic machinery that can result in photoinhibition [19]. F_v_/F_m_ decreased in heat sensitive cultivars of wheat, tomato (*Solanum lycopersicum*) and common beans (*Phaseolus vulgaris*) exposed to high temperatures; whereas heat tolerant cultivars of the same species showed no decrease in F_v_/F_m_, or quickly recovered after being exposed to a high temperature period [20,35,36,37,38]. In quinoa, F_v_/F_m_ decreased under drought stress [39] but did not under heat stress (40 °C) [37]. An increase in F_v_/F_m_ has been reported before for quinoa under high heat and drought exposure [30]. Although it is assumed that non-stressed F_v_/F_m_ values are approximately 0.83 for many species, the 10 genotypes evaluated in this study had a mean of 0.734 ± 0.001 under control conditions. The observed increase in F_v_/F_m_ in plants exposed to heat treatment indicates that a higher efficiency of electron transport is followed by a subsequent increase in gas exchange at higher temperatures [37]. This suggests that no heat induced damage occurred that could cause chronic photoinhibition which would result in a persistent lower F_v_/F_m_ rate [12]. F_v_/F_m_ was higher at pre-dawn than in the afternoon, with an average of 0.72 ± 0.002 for pre-dawn and 0.69 ± 0.004 for the afternoon. This is expected and may be due to transient damage to PSII during the day. The higher F_v_/F_m_ under the heat treatment reflects an increased capacity for electron transport and was supported by changes in the relative chlorophyll content and sustained integrity of PSII; similar observations have been reported [29,40,41,42].

Nighttime respiration is typically associated with maintenance and repair processes of PSII, although these processes are not evenly distributed through the day and night [43,44]. In our study, R_N_ was lower in the heat treatment than the control. This is in contrast to other species, which show increases in R_N_ as part of stress responses, such as in rice [44]. However, quinoa exposed to heat and salinity stress showed no difference in R_N_ [30,31]. A possible explanation for the reduced R_N_ we observed in the heat treatment group is lower assimilate availability [44,45] or a capacity of quinoa to acclimate its R_N_. As leaves acclimated to high temperatures improved their photosynthetic capacity, other metabolic processes such as leaf nitrogen assimilation and amino acid synthesis, mostly performed during the day, may have also increased, resulting in a higher demand for assimilates and day respiration.

To our knowledge, this is the first study to expose quinoa to temperatures of 45 °C for a four-day period to evaluate leaf physiological performance. Other studies combined high heat with other stressors (e.g., drought), whereas ours looked solely at high temperatures, which allowed us to conclude that high temperatures alone do not impair leaf physiological performance and C assimilation. Our study showed no differences in shoot and seed biomass, except on Quinhua which showed a trend (*p* = 0.06) towards lower seed biomass in the heat treatment, supporting our conclusion that a ‘heat wave’ by itself is not a major stressor for quinoa in the long term. Yet, under field conditions, it is common for plants to experience more than one stress at a time, underlining the importance to identify appropriate management techniques (e.g., supplemental irrigation) that could help mitigate the negative effects of compounded drought and heat on yield.

## 4. Materials and Methods

### 4.1. Plant Material and Growing Conditions

Experiments were conducted in a greenhouse and growth chambers at the Nevada Agricultural Experimental Station in the University of Nevada, Reno. Ten quinoa genotypes contrasting in leaf greenness index and seed biomass response to high temperatures were provided by Washington State University (Table 1) [26]. Plants were grown in 34.3 cm tall and 10.2 cm wide pots (2.65 L; ID# CP413CH; Stuewe & Sons, OR, USA) with a 1:1 mix of sand (Commercial Grade Quikrete 30 grit) and soil medium (Sungro Fafard^®^ 3B Mix, Metro-Mix^®^ 830). Prior to planting, the soil mix was watered to maximum water holding capacity, and seeds were planted approximately 0.6 cm deep. Plants were irrigated daily to full saturation by an automated system, with irrigation time increasing as plants became larger; pots were free to drain excess water. Plants were fertilized either with a 20-20-20 (Jack’s Fertilizer, J.R. Peters, Inc., PA) three times a week or with Osmocote (13-13-13) and Micromax Micronutrients applied at approximately 15–18 g (1/2 tablespoon), and 3–4 g (1/2 teaspoon) per pot, respectively. Plants received a 14 h photoperiod and supplemental light was used when needed. The greenhouse was kept at a temperature of 21 ± 6 °C and 17 ± 4 °C (mean ± standard deviation) during the day and night, respectively. Relative humidity was maintained between 35 and 45%. The heat treatment was applied once plants reached the sixth-leaf growth stage, approximately 6–8 weeks after planting, according to the BBCH scale [46], where anthesis had just begun and anthers were extruding; this is a growth stage known to be especially susceptible to heat stress that can result in reduced grain yield [10].

Conviron growth chambers (Model A1000 using the CMP6010 control system) were used to control temperature and light intensity for the duration of the simulated heat wave. Light intensity changed in three steps (400, 750 and 1050 mmol m^−2^ s^−1^) over the course of three hours to simulate sunrise and sunset in field conditions. Fans and vents were set to remove humidity from the chambers; relative humidity was 60–70% at night and 70–80% during the day for the control treatment, and 30–40% at night and 60–70% during the day for the heat treatment. The temperature regime is shown in Table 2.

### 4.2. Leaf Gas Exchange and Chlorophyll Fluorescence

Leaf gas exchange was measured for four consecutive days on the same fully mature leaf, towards the apical end of the plant, with a field portable open flow infrared gas analyzer (model 6400, LI-COR Inc., NE, USA). The area of the chamber was 6 cm^2^ and the middle portion of the leaf was used for measurements. Measurements were taken between 14:00 and 17:00, corresponding to times of potential maximum stress after two hours of exposure to high temperatures (Table 2). The photosynthetic photon flux density (PPFD) was set to 2000 μmol m^−2^ s^−1^, the reference CO_2_ concentration was set to 400 μmol CO_2_ mol^−1^, the flow was set to 500 μmol s^−1^, and the temperature of the block was set to 20 °C and 40 °C depending on whether the plant was subjected to a control or a heat temperature treatment. The parameters of interest were: photosynthetic rate (P_n_), stomatal conductance (g_s_) and intrinsic water use efficiency (WUE_i_: P_n_/g_s_).

Nighttime measurements to quantify dark respiration were taken two hours after sunset. The LI-6400 had a similar set up as described above with the exception that the photosynthetic photon flux density (PPFD) was set to 0 μmol m^−2^ s^−1^, the flow was reduced to 300 μmol s^−1^ or less, and the block temperature was set to <15 °C or 35 °C, corresponding to the control and heat temperature treatment, respectively. Nighttime respiration (R_N_) was measured as the absolute value of C assimilation (i.e., P_n_).

Chlorophyll fluorescence measurements were taken at pre-dawn, between 04:30 and 06:30, as well as in the afternoon between 14:00 and 16:00 using a modulated fluorometer (MultispeQ v1.0, PhotosynQ LLC., East Lansing, MI, USA). Measurements were conducted on the same leaf during the duration of the heat treatment, and leaves were fully mature and towards the apical end of the plant (i.e., same criteria as for leaf gas exchange).

### 4.3. Aboveground Biomass and Seed Weight

Plants were allowed to reach maturity in the greenhouse and water was shut down when seeds were formed in the panicles. After plants dried down in the greenhouse at ambient temperatures, all aboveground biomass (shoots and seeds) was oven dried at 60 °C for at least 48 h. Seed biomass was determined by threshing quinoa panicles, cleaning the seeds, and weighing them.

### 4.4. Data Processing and Analysis 

Trials were conducted in a randomized complete block design and analyzed using R version 3.5.1 (R Core Team, 2018). All ten genotypes were evaluated in every trial, which consisted of either 60 or 80 plants (i.e., 3 to 4 replicates per genotype by temperature treatment combination). Each response variable (e.g., P_n_, g_s_, F_v_/F_m_) was measured in at least three separate trials. A mixed effects modelling approach with restricted maximum likelihood (REML) was used, including *lme4* [47] nlme [48] and emmeans [49] packages. Genotype, treatment and their interaction (GxT) were considered fixed effects, whereas block, experiment and day were considered random effects. Model selection was performed on models that did not fail to converge using Akaike Information Criteria (AIC) values. Data were evaluated for normality based on visual inspection of residuals, and for homogeneity of variance using Levene’s Test in the car package [50]. When necessary, outliers were removed using Mahalanobis’s distance using mvoutlier [51]. The data was transformed when ANOVA assumptions were not met. Genotype and treatment means were compared using Tukey’s post hoc test with the emmeans package and significance assessed at *p* < 0.05 [30]. Data were organized and visualized using the dplyr package [52] and ggplot2 [53].

## Figures and Tables

**Table 1:** List of the ten quinoa genotypes used in this study with available information on plant introduction number (#) and geographical origin (Location).

Genotype	Plant Introduction #	Location
3UISE	AMES 13756	New Mexico, USA
UDEC-1	PI 634923	Bucalemu, Chile
QQ065	PI 614880	Los Lagos, Chile
Quinhua	N/A	Chile
17GR	AMES 13735	New Mexico, USA
QQ74	PI 614886	Maule, Chile
Pison	AMES 13746	New Mexico, USA
Titicaca	N/A	Denmark
Kaslaea	AMES 13745	New Mexico, USA
Japanese Strain	PI 677100	Washington, USA

**Table 2:** Temperature regime for 10 quinoa genotypes exposed to high temperature (heat treatment) and control treatment for a four-day simulated heat wave in growth chambers.

Time of Day	Treatment (°C)	
	Heat	Control
00:00	30	14
06:00	32	16
08:00	35	18
10:00	40	18
12:00	45	20
16:00	40	18
18:00	35	16
20:00	32	16
22:00	30	14

## Supplementary Materials

The following are available online at https://www.mdpi.com/2223-7747/9/1/81/s1, Figure S1: Relative chlorophyll by day of 10 quinoa genotypes exposed to a four-day heat treatment (**A**) (45 °C/30 °C) and control treatment (**B**) (20 °C/14 °C); day and night, respectively. Each point represents a mean relative chlorophyll content for each genotype (*n* = 5–9). The fitted lines indicate the slope calculated from the linear regression model where average relative chlorophyll is a response to daytime.
