# Supplementary material for: Leaf Gas Exchange Performance of Ten Quinoa Genotypes under a Simulated Heat Wave
Source: Plants (Basel). 2020 Jan 9;9(1):81. doi: 10.3390/plants9010081 (PMC7020487; doi:10.3390/plants9010081)
Supplement: Supplementary file 1 [file plants-09-00081-s001.pdf]

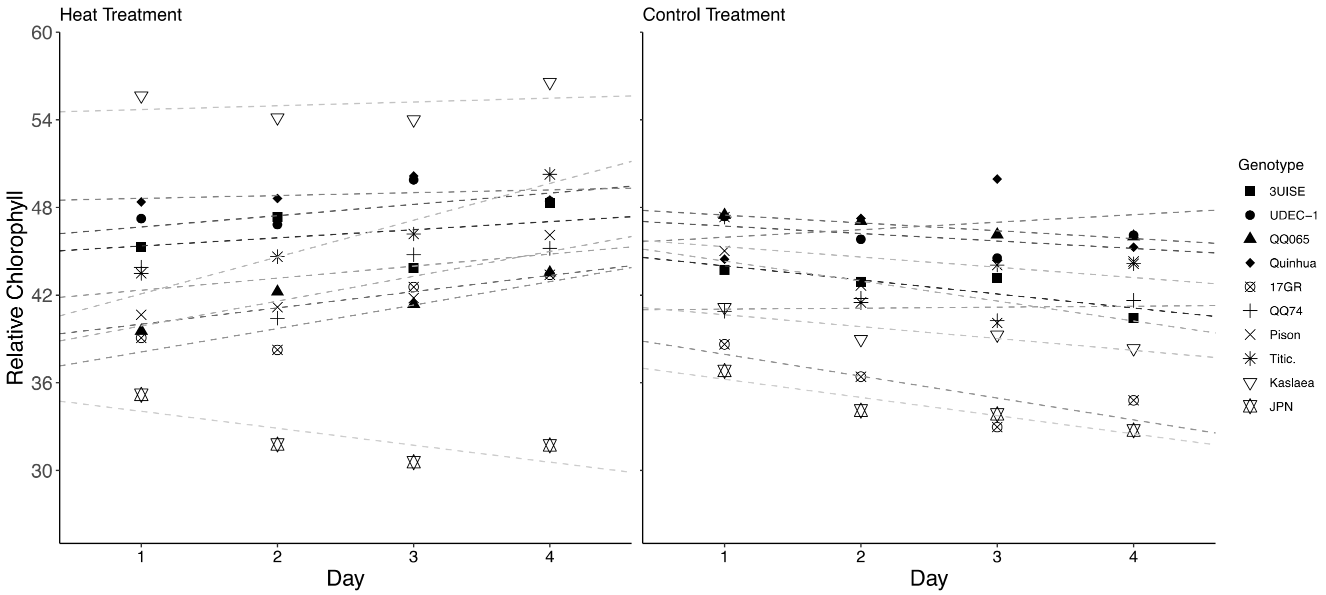

Figure S1. Relative chlorophyll by day of 10 quinoa genotypes exposed to a four-day heat treatment (**A**) (45 °C/30 °C) and control treatment (**B**) (20°C/14°C); day and night, respectively. Each point represents a mean relative chlorophyll content for each genotype (*n* = 5–9). The fitted lines indicate the slope calculated from the linear regression model where average relative chlorophyll is a response to daytime.
